# Imbalance of Bone Homeostasis Caused by Nrf2 Deficiency Leads to Bone Loss in OVX Rats

**DOI:** 10.1155/sci/7214250

**Published:** 2025-08-28

**Authors:** Pan Sun, Zhiqiang Wang, Sainan Chen, Xuzheng Chen, Fen Zhou, Chutian Zhang, Juan Yang, Yunmei Huang, Yanping Lin

**Affiliations:** ^1^Shanghai Municipal Hospital of Traditional Chinese Medicine, Shanghai University of Traditional Chinese Medicine, Shanghai 200071, China; ^2^Academy of Integrative Medicine, Fujian University of Traditional Chinese Medicine, Fuzhou 350122, Fujian, China; ^3^Fujian Key Laboratory of Integrative Medicine on Geriatrics, Fuzhou 350122, Fujian, China; ^4^College of Acupuncture and Moxibustion, Fujian University of Traditional Chinese Medicine, Fuzhou 350122, Fujian, China

**Keywords:** bone homeostasis, bone loss, nrf2, postmenopausal osteoporosis, ROS

## Abstract

**Objective:** Postmenopausal osteoporosis (PMOP) is a common bone metabolic disorder in middle-aged and elderly women, yet its pathogenesis remains unclear. This study investigates the effect of nuclear factor erythroid 2–related factor 2 (Nrf2) deficiency on bone homeostasis to provide insight into the mechanisms underlying PMOP.

**Methods:** Sixteen female SD rats were randomly assigned to Sham and ovariectomized (OVX) groups. After 12 weeks, bone homeostasis disruption and Nrf2-mediated oxidative stress responses in bone tissue cells were assessed. Nrf2 expression was modulated in UMR-106 osteoblast-like cells and RAW264.7 macrophage-derived osteoclast precursor cells through knockdown or pharmacological activation. The effects on osteogenic function and osteoclast differentiation under oxidative stress were then evaluated.

**Results:** The OVX group of rats exhibited a disruption in bone homeostasis, potentially attributable to the reduced expression of Nrf2 and its downstream antioxidant enzymes, coupled with elevated levels of oxidative stress. Nrf2 knockdown impaired osteogenic capacity in UMR-106 cells and enhanced osteoclast differentiation in RAW264.7 cells. In contrast, activation of Nrf2 using tert-butylhydroquinone (TBHQ) promoted bone formation and suppressed osteoclast differentiation and bone resorption.

**Conclusion:** Nrf2 deficiency may contribute to PMOP by disrupting bone homeostasis. Activation of Nrf2 may represent a potential therapeutic strategy for restoring bone balance and treating PMOP.

## 1. Introduction

Postmenopausal osteoporosis (PMOP) is a metabolic bone disease commonly observed in middle-aged and elderly women after menopause. It is characterized by rapid loss of bone mass, decreased bone strength, altered bone microstructure, and an increased risk of fractures. The bone remodeling process is essential for maintaining the physiological function of the skeleton, ensuring normal mineralization, and repairing micro-damage and micro-fractures [1–3]. This process requires a balance between osteoblast-mediated bone formation and osteoclast-mediated bone resorption, a dynamic equilibrium referred to as bone homeostasis. Osteoblasts and osteoclasts are regulated by various hormones, growth factors, and signaling pathways to sustain bone homeostasis [4–6]. Disruption of this equilibrium can result in metabolic bone diseases, including osteoporosis and osteosclerosis. Although the precise pathogenesis of PMOP remains unclear, it is widely accepted that a rapid decline in estrogen levels after menopause leads to loss of bone homeostasis, with bone resorption exceeding bone formation. Therefore, correcting this imbalance and restoring bone homeostasis is critical for the treatment of PMOP. However, the mechanisms that govern this imbalance remain incompletely understood.

Reactive oxygen species (ROS) are molecules produced during oxygen metabolism that contain unpaired electrons. Under physiological conditions, antioxidant enzymes scavenge excess ROS. However, excessive accumulation of ROS or insufficient synthesis of antioxidant enzymes disrupts the balance between oxidative stress and antioxidant defense, causing cellular damage to lipids, proteins, and nucleic acids [7, 8]. Recent studies indicate that oxidative stress plays a significant role in the pathogenesis of various diseases [9, 10], including osteoporosis [11, 12]. Oxidative stress can downregulate osteogenic genes such as RUNX2 and Osterix by influencing protein kinases, including MAPKs, resulting in decreased osteoblast activity [13, 14]. It can also alter the osteoprotegerin (OPG)/RANK/RANKL signaling pathway, leading to reduced OPG expression and enhanced osteoclast differentiation and function [15–17]. In ovariectomized (OVX) rats, levels of antioxidant enzymes such as catalase (CAT) and superoxide dismutase (SOD) decline, promoting bone resorption over bone formation. Estrogen supplementation increases antioxidant enzyme activity and attenuates bone loss [18].

The Nrf2 signaling pathway plays a central role in the cellular defense against oxidative stress. Nuclear factor erythroid 2-related factor 2 (Nrf2), a member of the cap-n-collar (CNC) transcription factor family, contains six highly conserved domains (Nrf2 ECH homology, Neh1–6). The Neh1 domain includes a C-terminal leucine zipper (bZIP) structure, enabling heterodimerization with small Maf proteins and recognition of antioxidant response elements (AREs) [19]. The Neh2 domain mediates Nrf2 interaction with its negative regulator KEAP1. Under basal conditions, Nrf2 is primarily bound to and degraded by KEAP1, resulting in low cytoplasmic levels [19–21]. Upon increased cellular ROS, protein kinases facilitate the dissociation of Nrf2 from KEAP1, allowing Nrf2 to translocate into the nucleus. There, it binds to AREs and promotes the transcription and translation of antioxidant enzymes [20, 21]. Studies suggest that Nrf2 deficiency in female mice leads to decreased bone mass and accelerated bone loss, whereas Nrf2 activation reduces oxidative stress and is effective in treating osteoporosis [22, 23]. Nevertheless, the specific regulatory mechanisms of Nrf2 in bone homeostasis remain unclear and require further investigation.

In this study, oxidative stress in osteoblasts and osteoclasts was induced using hydrogen peroxide to investigate the specific role of Nrf2 in these cell types. The findings show that Nrf2 reduces oxidative stress in osteoblasts and promotes their differentiation. In osteoclasts, Nrf2 reduces oxidative stress and inhibits their differentiation and bone resorption. These results provide insight into the pathogenesis of PMOP and identify Nrf2 as a potential therapeutic target for treatment.

## 2. Materials and Methods

### 2.1. Regents

The bone alkaline phosphatase (BALP) and tartrate-resistant acid phosphatase (TRACP)-5b ELISA kits were obtained from CUSABIO (China). Antibodies against Nrf2, KEAP1, HO-1, RUNX2, NFATc1, β-actin, and TBP were sourced from PROTEINTECH (USA). The phospho-Nrf2 antibody was acquired from AFFINITY BIOSCIENCES (USA). The TRACP staining kit and the malondialdehyde (MDA), total SOD (T-SOD), CAT, and glutathione peroxidase (GSH-PX) assay kits were purchased from Nanjing Jiancheng Bioengineering Institute (China). The dihydroethidium (DHE) probe was obtained from BEYOTIME in China.

### 2.2. Animal Experiment

The Ethics Committee of Fujian University of Traditional Chinese Medicine approved this study (No. 2018038), and all animal procedures complied with the Experimental Animal Ethics Method of Fujian University of Traditional Chinese Medicine. Sixteen healthy female specific-pathogen-free (SPF) rats, aged 3 months, were purchased from the Experimental Animal Center of Zhejiang Province (Number 1810100034). Rats were housed in a controlled laboratory environment with 60% relative humidity, a temperature of 25°C, and a 12-h light/dark cycle. All animals received pathogen-free feed and water and were allowed to acclimate for 1 week before random allocation to two groups: eight rats underwent bilateral ovariectomy (OVX group), and eight rats underwent a sham operation involving removal of an equivalent amount of peri-ovarian fat. Following 12 weeks of standard feeding after surgery, all animals were sacrificed. Femurs, tibias, lumbar vertebrae, and serum samples were collected. In order to alleviate the pain of animals, rats were anesthetized with an intraperitoneal injection of 2% pentobarbital sodium (40 mg/kg; Sigma–Aldrich) during model establishment and euthanasia. Blood was collected from the abdominal aorta, left to stand at room temperature for 3–4 h, and centrifuged at 3000 rpm for 15 min. The supernatant was separated and aliquoted. Surrounding muscle was removed from the femur, tibia, and lumbar vertebrae, and bone tissues were preserved according to experimental requirements.

### 2.3. Cell Culture

UMR-106 and RAW264.7 cells were obtained from the Cell Bank of the Chinese Academy of Sciences. Cells were cultured in high-glucose DMEM supplemented with 10% fetal bovine serum (Gibco, USA) and 1% penicillin–streptomycin (HyClone, New Zealand) in a 5% CO_2_ humidified incubator at 37°C. Oxidative stress was induced using 10 μM H_2_O_2_ (Sinopharm, China). Osteoclast differentiation in RAW264.7 cells was induced by 50 ng/mL RANKL (PeproTech, USA).

### 2.4. Lentivirus Transfection

UMR-106 cells were seeded in 12-well plates at 5 × 10^4^ cells per well. After 24 h, transfection was conducted using LV-Nrf2-RNAi lentivirus (MOI 50; GeneChem, China). After 12 h, the medium was replaced with conventional culture medium. At 72 h post-transfection, selection was performed with 3 μg/mL puromycin (Beyotime, China), and the medium was refreshed every 3 days. After 1 week, the puromycin concentration was reduced to 1.5 μg/mL. After another week, cell RNA was extracted for verification of transfection efficiency. RAW264.7 cells were plated and transfected using LV-Nrf2-RNAi lentivirus (MOI 20) under the same procedure, with puromycin selection at 4 μg/mL for 1 week and 2 μg/mL for the subsequent week. Cell RNA was then extracted for verification.

### 2.5. Bone Quality and Bone Strength Measurement

Bone mineral density (BMD) of the right femur was measured using dual-energy X-ray absorptiometry (Hologic, USA). The maximum load of the tibia was assessed using a three-point bending test and vertical compression test with a universal testing machine (IG-A1000N; Shimadzu, Japan). The maximum load of three lumbar vertebrae was also measured.

### 2.6. Histopathological Analysis

The proximal tibia was fixed in 4% paraformaldehyde, followed by decalcification in EDTA solution for 3 months. Bone tissues were dehydrated and embedded in paraffin. Sections of 4 μm thickness were prepared and stained using Masson's trichrome to evaluate pathological changes.

### 2.7. ELISA

The ELISA kit was equilibrated to room temperature for 30 min before use. Standards and test samples (100 μL each) were added to their respective wells. Plates were sealed and incubated at 37°C. After discarding the liquid, 100 μL of antibody working solution was added to each well and incubated at 37°C for 60 min. Plates were washed three times, then the ABC solution was added and incubated for 30 min at 37°C. After five additional washes, TMB substrate was added for color development in the dark at 37°C. The stop solution was added to terminate the reaction. Optical density values were measured using an ELx800 microplate reader (BioTek, USA), and sample concentrations were calculated accordingly.

### 2.8. MDA and Antioxidant Enzyme

A portion of the femur was ground to prepare a 10% bone tissue homogenate. The concentrations of MDA, T-SOD, CAT, and GSH-PX in the homogenate were measured according to the kit instructions. Absorbance was read using an ELx800 microplate reader (BioTek, USA).

### 2.9. Western Blot

Bone tissue or cells were lysed in RIPA buffer (Beyotime, China) containing phosphatase inhibitor and PMSF for 30 min. Lysates were centrifuged at 14,000 rpm for 20 min, and the supernatant was collected. Protein concentration was quantified using the BCA method. Each sample (100 μL) was mixed with 25 μL loading buffer and denatured at 95°C for 5 min. Proteins (20 μg per lane) were separated by SDS–PAGE at 20 V for 10 min, 50 V for 40 min, and 120 V for 60 min, then transferred to a 0.2 μm PVDF membrane at 100 V for 120 min. Membranes were blocked with skim milk for 60 min, washed three times with TBST, and incubated with primary antibody (1:1000) at 4°C overnight. After washing, membranes were incubated with secondary antibody at room temperature for 60 min, washed again, and developed for protein detection.

### 2.10. Alizarin Red S Staining

UMR-106 cells were cultured for 14 days, washed three times with PBS, and fixed with 4% paraformaldehyde for 30 min. Cells were again washed three times with PBS, stained with Alizarin Red S solution at 37°C for 20 min, rinsed with PBS, and examined microscopically. Quantitative analysis of ARS was performed using Image Pro Plus 6.0.

### 2.11. Quantitative RT-PCR

Total RNA was extracted from cells using TRIzol reagent. For cDNA synthesis, 1 μg RNA was mixed with 2 μL gDNA Remover, 5 μL 4 × Master Mix, and 12 μL ddH_2_O, followed by reverse transcription. Use the primers (designed and synthesized by Shanghai Sangon Biotech) listed in Table 1 to detect the mRNA expression levels of Nrf2, ALP, osteocalcin (OCN), osteopontin (OPN), bone sialoprotein (BSP), TRACP, and CTSK through real-time fluorescence quantitative PCR. Quantitative PCR was performed using a 2 × Color SYBR Green qPCR Master Mix kit (EZBioscience, USA) in a 20 μL reaction volume. Relative gene expression was calculated according to kit instructions.

### 2.12. DHE

After experimental intervention, UMR-106 and RAW264.7 cells were washed three times with PBS and fixed in 4% paraformaldehyde for 30 min. The DHE probe was diluted with PBS to 2 μM, and 1 mL was added per dish. Dishes were incubated at 37°C for 30 min, then washed three times with PBS. DAPI staining solution (500 μL) was added for 5 min at 37°C, followed by further washing. Cells were observed and imaged with a confocal laser scanning microscope. DHE red fluorescence (561 nm) indicated intracellular superoxide anions; DAPI blue fluorescence (488 nm) marked nuclei. Relative fluorescence intensity was analyzed using ImageJ.

### 2.13. TRACP Staining

RAW264.7 cells, after 7 days of differentiation, were washed three times with PBS and fixed in 4% paraformaldehyde for 30 min. After washing, 1 mL of TRACP staining solution was added per well, and plates were incubated at 37°C in the dark for 1 h. After staining, wells were washed three times with PBS and counterstained with hematoxylin for 2 min, followed by additional PBS washes. The number of TRACP-positive multinucleated cells was assessed microscopically.

### 2.14. Statistical Analysis

Data were analyzed using SPSS 23.0 software and are presented as mean ± standard deviation ($\overset{―}{x}$ ± *S*). Student's *t*-test was applied for comparisons between groups, with *p* < 0.05 considered statistically significant.

## 3. Results

### 3.1. OVX Rats Exhibited Bone Loss and Imbalanced Bone Homeostasis

**Figure 1:**
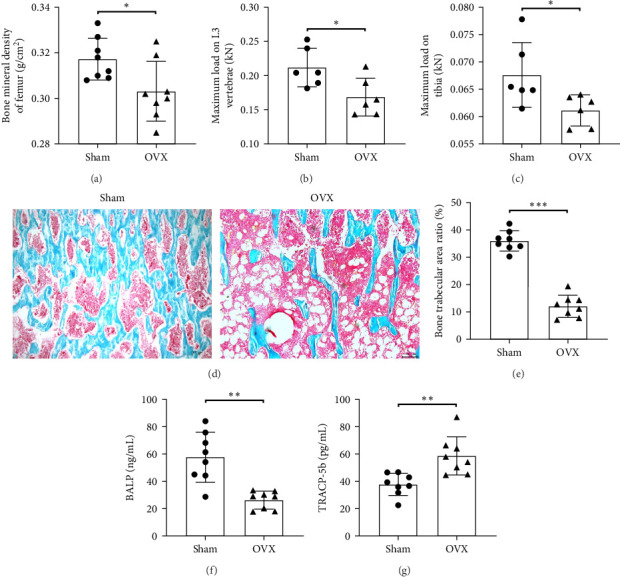
OVX rats exhibited bone loss and imbalanced bone homeostasis. (A) Femur bone density; (B) tibia maximal load; (C) L3 vertebral maximal load; (D, E) tibial cortical bone relative area of trabecular bone (scale bar: 100 μm); (F) serum bone alkaline phosphatase; and (G) serum tartrate-resistant acid phosphatase-5b. The data are presented as mean ± SD (Student's *t*-test, 6–8 independent samples). *⁣*^*∗*^*p* < 0.05, *⁣*^*∗∗*^*p* < 0.01, *⁣*^*∗∗∗*^*p* < 0.001.

The OVX model is widely used to study osteoporosis. As shown in Figure 1A–C, OVX rats display a pronounced reduction in bone density and biomechanical strength. There is a decrease in trabecular number and an increase in trabecular disruption, resulting in wider intertrabecular spaces (Figure 1D,E). The bone formation marker BALP is significantly reduced in the OVX group (Figure 1F), whereas the bone resorption marker TRACP-5b is significantly elevated (Figure 1G), indicating a loss of bone homeostasis following ovariectomy.

### 3.2. Nrf2 and Antioxidant Enzymes Decreased in the Bone Tissue of OVX Rats

**Figure 2:**
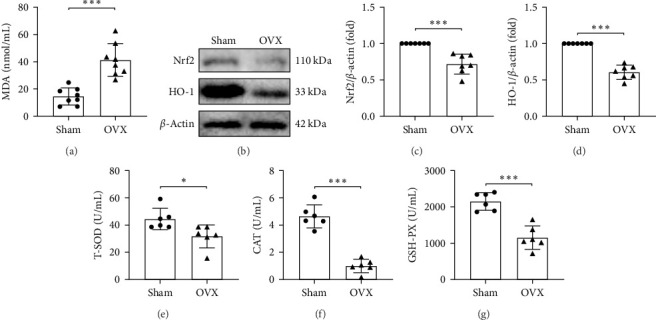
Nrf2 and antioxidant enzymes decreased in the bone tissue of OVX rats. (A) MDA content in bone tissue; (B–D) relative expression levels of Nrf2 and HO-1 in bone tissue; and (E–G) T-SOD, CAT, and GSH-PX levels in bone tissue. The data are presented as mean ± SD (*n* = 6–7 independent samples, Student's *t*-test). *⁣*^*∗*^*p* < 0.05, *⁣*^*∗∗∗*^*p* < 0.001.

ROS can react with unsaturated fatty acids in biological membranes, leading to the formation of lipid peroxides such as MDA [24]. MDA content is significantly increased in the bone tissue of OVX rats (Figure 2A), indicating oxidative stress after ovariectomy. Western blot analysis shows a reduction in Nrf2 (Figure 2B,C) and its downstream antioxidant enzymes, including HO-1, T-SOD, CAT, and GSH-Px, compared to the Sham group (Figure 2B,D–G). The observed increase in MDA, together with reduced Nrf2 and antioxidant enzymes, indicates that oxidative stress in bone tissue is associated with inhibition of the Nrf2 signaling pathway.

### 3.3. Nrf2 Deficiency Exacerbates Oxidative Stress and Inhibits Osteoblast Function

**Figure 3:**
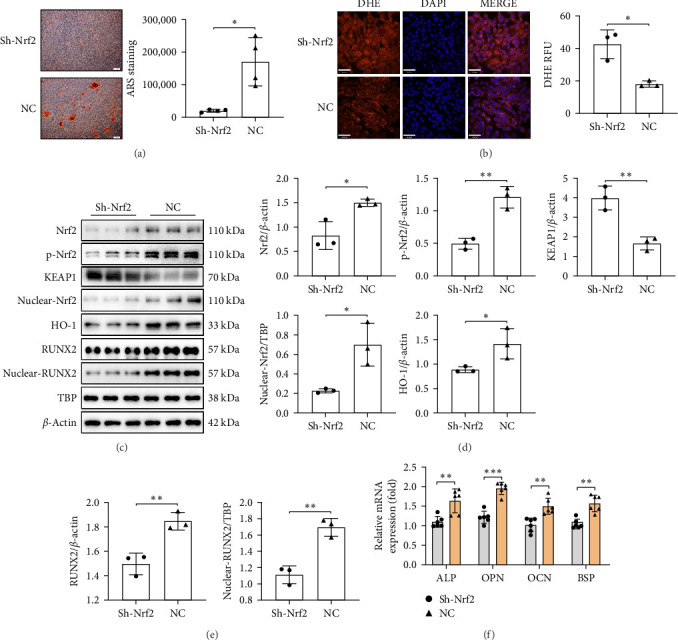
Nrf2 deficiency exacerbates oxidative stress and inhibits osteoblast function. (A) Mineralization ability of UMR-106 cells after Nrf2 gene knockdown (scale bar: 100 μm); (B) ROS content in UMR-106 cells after Nrf2 knockdown (scale bar: 70 μm); (C–E) relative expression of Nrf2, p-Nrf2, KEAP1, nuclear Nrf2, HO-1 (D), RUNX2 and nuclear RUNX2 (E) in UMR-106 cells after Nrf2 knockdown; and (F) relative mRNA levels of bone formation-related genes ALP, OPN, OCN, and BSP. The data are presented as mean ± SD (Student's *t*-test, 3–6 independent samples). *⁣*^*∗*^*p* < 0.05, *⁣*^*∗∗*^*p* < 0.01, *⁣*^*∗∗∗*^*p* < 0.001.

To investigate the effect of Nrf2 on osteoblast function under oxidative stress, Nrf2 gene expression in UMR-106 cells was knocked down using lentiviral transfection. Mineralization capacity decreased significantly (Figure 3A), whereas intracellular ROS increased (Figure 3B) after Nrf2 knockdown. Western blot analysis revealed lower levels of both Nrf2 and phospho-Nrf2, accompanied by increased KEAP1. Therefore, nuclear Nrf2 content was reduced, leading to decreased expression of the antioxidant enzyme HO-1 and suppression of the Nrf2 signaling pathway (Figure 3C,D). The content of the transcription factor RUNX2 was also significantly reduced in both cytoplasmic and nuclear fractions (Figure 3C,E). Furthermore, mRNA levels of bone formation-associated genes ALP, OPN, OCN, and BSP were significantly decreased (Figure 3F), indicating that osteoblast function was suppressed.

### 3.4. Nrf2 Deficiency Exacerbates Oxidative Stress and Promotes Osteoclast Differentiation

**Figure 4:**
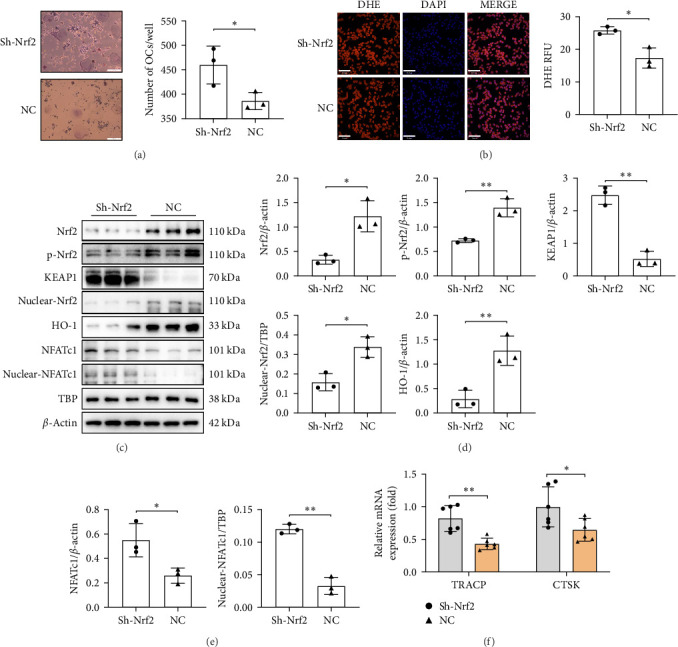
Nrf2 deficiency exacerbates oxidative stress and promotes osteoclast differentiation. (A) RAW264.7 cell differentiation toward osteoclasts after Nrf2 gene knockdown (scale bar: 200 μm); (B) ROS content in RAW264.7 cells after Nrf2 knockdown (scale bar: 70 μm); (C–E) relative expression of Nrf2, p-Nrf2, KEAP1, nuclear Nrf2, HO-1 (D), NFATc1 and nuclear NFATc1 (E) in RAW264.7 cells after Nrf2 knockdown; and (F) relative mRNA levels of bone resorption-related genes TRACP and CTSK. The data are presented as mean ± SD (Student's *t*-test, 3–6 independent samples). *⁣*^*∗*^*p* < 0.05, *⁣*^*∗∗*^*p* < 0.01.

To investigate the impact of Nrf2 on osteoclast differentiation under oxidative stress, RAW264.7 cells were transfected with a lentivirus to suppress the expression of the Nrf2 gene. The outcomes revealed a significant increase in the differentiation of RAW264.7 cells into osteoclasts (Figure 4A) and the content of intracellular ROS (Figure 4B) after the knockdown of Nrf2. Additionally, Western blot results indicated a decrease in both Nrf2 and phospho-Nrf2 levels, alongside an increase in KEAP1. Consequently, the nuclear content of Nrf2 was also decreased, leading to a reduction in the antioxidant enzyme HO-1 and the inhibition of the Nrf2 signaling pathway (Figure 4C,D). Furthermore, the transcription factor NFATc1 content in osteoclasts increased significantly both in the cells and nucleus (Figure 4C,E), and the expression of bone resorption-related genes TRACP and CTSK mRNA was significantly increased (Figure 4F).

### 3.5. Activation of Nrf2 Alleviates Oxidative Stress and Restores Osteoblast Function

**Figure 5:**
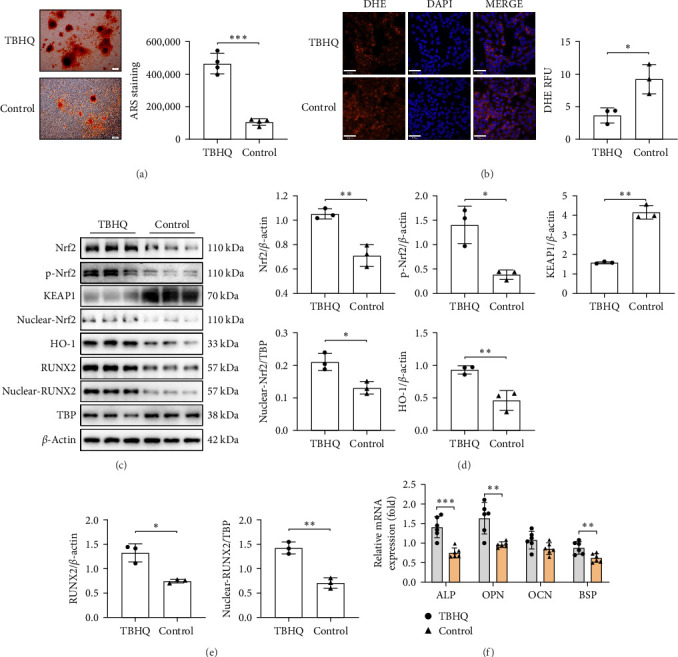
Activation of Nrf2 alleviates oxidative stress and restores osteoblast function. (A) Mineralization ability of UMR-106 cells after TBHQ activates Nrf2 (scale bar: 100 μm); (B) ROS content in UMR-106 cells after TBHQ activation (scale bar: 70 μm); (C–E) Relative expression of Nrf2, p-Nrf2, KEAP1, nuclear Nrf2, HO-1 (D), RUNX2, and nuclear RUNX2 (E) in UMR-106 cells after TBHQ activation; and (F) relative mRNA levels of bone formation-related genes ALP, OPN, OCN, BSP. The data are presented as mean ± SD (Student's *t*-test, 3–6 independent samples). *⁣*^*∗*^*p* < 0.05, *⁣*^*∗∗*^*p* < 0.01, *⁣*^*∗∗∗*^*p* < 0.001.

To further clarify the role of Nrf2 in bone tissue remodeling under oxidative stress, the Nrf2 activator TBHQ (2 μM, MCE, China) was applied to UMR-106 cells. The tert-butylhydroquinone (TBHQ) treatment resulted in a significant increase in mineralization (Figure 5A) and a decrease in cellular ROS (Figure 5B). TBHQ activated the Nrf2 pathway, promoting nuclear translocation of Nrf2 and upregulation of the antioxidant enzyme HO-1 (Figure 5C,D). RUNX2 expression in both cytoplasm and nucleus was increased (Figure 5C,E), and mRNA levels of bone formation-associated genes ALP, OPN, and BSP were significantly elevated (Figure 5F).

### 3.6. Activation of Nrf2 Alleviates Oxidative Stress and Inhibits Osteoclast Differentiation

**Figure 6:**
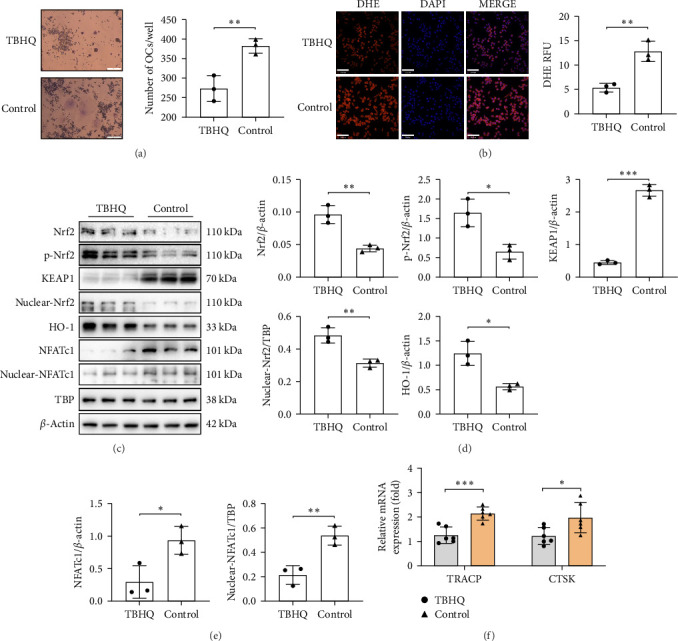
Activation of Nrf2 alleviates oxidative stress and inhibits osteoclast differentiation. (A) TBHQ-induced Nrf2 activation alters RAW264.7 cell differentiation toward osteoclasts (scale bar: 200 μm); (B) TBHQ-induced Nrf2 activation decreases ROS in RAW264.7 cells (scale bar: 70 μm); (C–E) relative expression of Nrf2, p-Nrf2, KEAP1, nuclear Nrf2, HO-1 (D), NFATc1, and nuclear NFATc1 (E) in RAW264.7 cells after TBHQ activation; and (F) relative mRNA levels of bone resorption-related genes TRACP and CTSK. The data are presented as mean ± SD (Student's *t*-test, 3–6 independent samples). *⁣*^*∗*^*p* < 0.05, *⁣*^*∗∗*^*p* < 0.01, *⁣*^*∗∗∗*^*p* < 0.001.

In RAW264.7 cells, TBHQ (2 μM) treatment led to a significant reduction in osteoclast differentiation (Figure 6A) and cellular ROS (Figure 6B). Activation of the Nrf2 pathway increased nuclear Nrf2 and HO-1 expression (Figure 6C,D). Both cytoplasmic and nuclear NFATc1 levels decreased significantly (Figure 6C,E), and mRNA expression of bone resorption-associated genes TRACP and CTSK was also significantly reduced (Figure 6F).

## 4. Discussion

The Nrf2 pathway is a well-known antioxidant stress signaling pathway with established roles in various diseases. However, its effect on osteoporosis, particularly in relation to bone homeostasis, remains less well understood. Further research is necessary to clarify the specific role of Nrf2 in this context.

BALP and TRACP-5b are established biomarkers reflecting osteoblast and osteoclast activity, respectively. Variations in serum levels of BALP and TRACP-5b indicate changes in bone homeostasis [25, 26] In this study, significant reductions in bone mass, strength, and trabecular number were observed in OVX rats, attributable to disrupted bone homeostasis.

Bone tissue in OVX rats experienced oxidative stress, as evidenced by increased MDA levels. MDA is produced when ROS react with unsaturated fatty acids in cell membranes. Elevated MDA levels indicated that bone tissue cells were under oxidative stress, which can impair the osteogenic differentiation and functional activity of BMSCs and osteoblasts [27, 28]. This results in decreased osteoblast number and function, reduced BALP secretion, and lower serum BALP. Osteoblast-secreted OPG can compete with RANKL for binding to RANK, preventing osteoclast differentiation. However, ROS inhibits osteoblast activity and OPG secretion, increasing the rate of RANKL–RANK binding, promoting osteoclast differentiation, and enhancing bone resorption [15–17]. These findings indicate that oxidative stress after ovariectomy disrupts bone homeostasis, resulting in bone resorption by osteoclasts exceeding bone formation.

The Nrf2 signaling pathway is critical for cellular resistance to oxidative stress. Upon nuclear translocation, Nrf2 induces transcription of antioxidant enzymes such as HO-1, SOD, GSH-PX, and CAT. These enzymes remove excess ROS, prevent oxidative stress damage, and help maintain antioxidant system balance. After ovariectomy, both Nrf2 and its downstream antioxidant enzymes are significantly decreased in bone tissue, indicating that the Nrf2 signaling pathway is in a “blocked” state after estrogen decline. This “blocked” state underlies the imbalance in the antioxidant system and the occurrence of oxidative stress, as Nrf2 is a central component of the antioxidant response.

To investigate the role of Nrf2 in bone homeostasis, Nrf2 gene expression was modulated in both osteoblasts and osteoclast precursor cells. Knockdown of Nrf2 in osteoblasts under oxidative stress resulted in reduced antioxidant enzyme synthesis and ROS accumulation, which impaired proliferation, differentiation, mineralization, and BALP synthesis [13, 14]. The suppression of osteoblast function aligns with the regulatory effect of runt-related transcription factor 2 (RUNX2) on bone formation-related proteins, including OCN, OPN, and BSP [29]. During osteoblast differentiation and bone matrix synthesis, RUNX2 translocates to the nucleus, binds gene promoters, and drives bone formation. Reduced Nrf2 expression decreased both cytoplasmic and nuclear RUNX2, as well as the expression of osteoblast-related genes (OCN, OPN, BSP, and ALP). These results indicate that Nrf2 deficiency-induced ROS accumulation inhibits osteoblast function by suppressing RUNX2 expression.

Osteoclasts dissolve bone tissue by releasing hydrogen ions and proteolytic enzymes into the extracellular space. This process requires substantial ATP production, and osteoclasts contain more mitochondria than any other cell type [30, 31]. Mitochondria are the main source of ROS. Previous work demonstrated that ROS, especially hydrogen peroxide, is essential in regulating osteoclast differentiation and bone resorption. Osteoblast-derived RANKL binds to RANK, activating TRAF6 and downstream MAPK pathways (ERK, p38, and JNK) [32, 33]. These activate NFATc1, which controls transcription of osteoclast differentiation-related genes such as TRACP and CTSK. ROS acts as a key secondary messenger in this process. The present study showed that Nrf2 knockdown accelerated RANKL-induced differentiation of osteoclast precursors, associated with increased ROS, enhanced NFATc1 activation, and nuclear translocation. These data suggest that Nrf2 deficiency contributes to bone homeostasis imbalance by promoting osteoclastogenesis.

TBHQ, an Nrf2 activator, was used to increase cellular ROS clearance by dissociating the KEAP1-Nrf2 dimer and upregulating Nrf2 [34, 35]. TBHQ-induced Nrf2 activation promoted ROS clearance and nuclear translocation of RUNX2 in osteoblasts, whereas inhibiting NFATc1 synthesis and nuclear translocation in preosteoclasts, thus suppressing osteoclast differentiation. TBHQ-mediated Nrf2 activation also enhanced osteogenic differentiation under cyclic mechanical stretching in periodontal ligament stem cells [36]. In osteoclasts, TBHQ-activated Nrf2 limited differentiation, reducing bone resorption and injury caused by prosthetic metal particles [37]. These findings highlight the role of Nrf2 activation in restoring balanced bone remodeling.

The results confirm that Nrf2 loss impairs both bone formation and resorption. Previous studies demonstrated that Nrf2 knockout mice exhibit significant bone loss. Nrf2 prevents mesenchymal stem cell (MSC) dysfunction during aging [38], supporting bone health and osteoblast function [39], and its absence affects osteoclast differentiation [40, 41]. Moreover, drugs that activate Nrf2 lose efficacy in Nrf2-deficient models [38, 39], indicating Nrf2 as a promising therapeutic target for bone loss.

**Figure 7:**
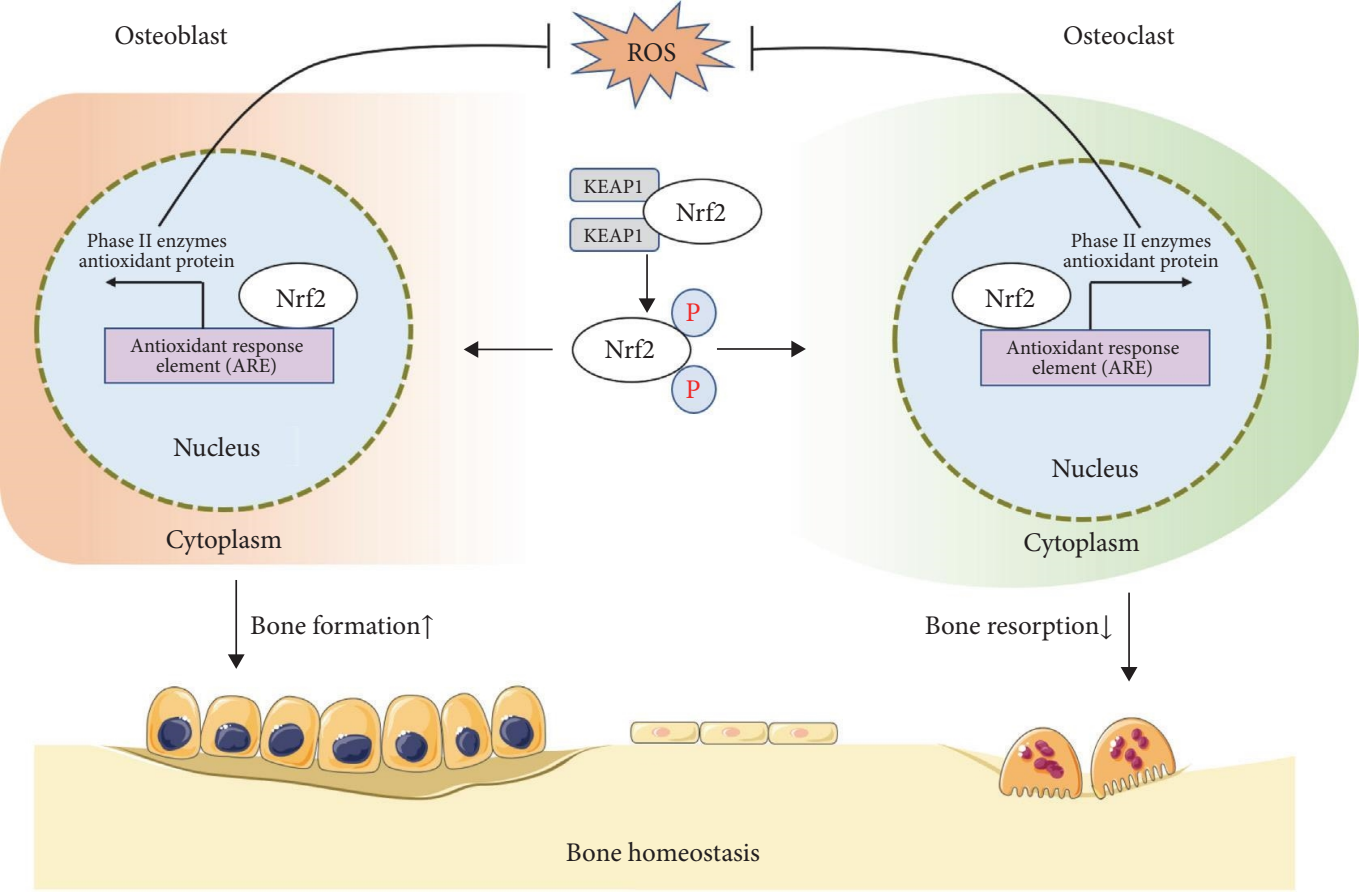
Mechanisms by which Nrf2 regulates bone homeostasis.Under oxidative stress, Nrf2 pathway activation promotes the expression of antioxidant enzymes that scavenge ROS. This process promotes osteoblast-mediated bone formation, inhibits osteoclast differentiation and bone resorption, and restores bone homeostasis.

In summary, regulation of Nrf2 in osteoblasts and osteoclasts demonstrates that Nrf2 promotes osteoblast bone formation, suppresses osteoclast differentiation and bone resorption, and restores bone homeostasis via modulation of oxidative stress (Figure 7).

## 5. Conclusion

This study confirms that Nrf2 deficiency disrupts bone homeostasis, which may contribute to the development of PMOP. Restoring bone homeostasis by activating the Nrf2 pathway could represent a therapeutic strategy for PMOP. However, the current research is limited by the absence of in vivo evidence regarding the specific role of Nrf2. Further clarification of the association between Nrf2 and bone homeostasis may be obtained by targeted deletion of the Nrf2 gene in osteoblasts and osteoclasts in vivo. Ongoing studies will further explore the mechanism by which Nrf2 regulates bone homeostasis.

## Figures and Tables

**Table 1:** Sequence of primers used in this study.

Gene	Primer sequences: *F* (5′–3′)	Primer sequences: *R* (5′–3′)
*Nrf2* (rat)	CGATTAGAGGCTCATCTCACAA	GTTGAATTGCTCCTTGGACATC
*ALP* (rat)	CGTTTTCACGTTTGGTGGCT	ACCGTCCACCACCTTGTAAC
*OCN* (rat)	AATAGACTCCGGCGCTACCT	ATAGATGCGCTTGTAGGCGT
*OPN* (rat)	GAGCAGTCCAAGGAGTATAAGC	AACTCGTGGCTCTGATGTTC
*GAPDH* (rat)	ACGGCAAGTTCAACGGCACAG	GAAGACGCCAGTAGACTCCACGAC
*BSP* (rat)	ACAACACTGCGTATGAAACCTATGAC	AGTAATAATCCTGACCCTCGTAGCC
*Nrf2* (mus)	ACACGAGATGAGCTTAGGGC	TCGGATCAATGCGAGCTGAG
*TRACP* (mus)	CAGCCAAGGAGGACTACGTT	CACATAGCCCACACCGTTCT
*CTSK* (mus)	AATTATGGCTGTGGAGGCGG	TGCATTTAGCTGCCTTTGCC
*GAPDH* (mus)	CATCACTGCCACCCAGAAGACT	CACTGACACATTGGGGGTAG

## Data Availability

The data that support the findings of this study are available from the corresponding author upon reasonable request.

## Nomenclature

PMOP: Postmenopausal osteoporosis
OVX: Ovariectomy
ROS: Reactive oxygen species
BMD: Bone mineral density
MDA: Malondialdehyde
T-SOD: Total superoxide dismutase
CAT: Catalase
GSH-PX: Glutathione peroxidase
BALP: Bone alkaline phosphatase
TRACP-5b: Tartrate-resistant acid phosphatase-5b
TBHQ: Tert-butylhydroquinone.
